# Prospective clinical study of enamel wear caused by monolithic zirconia resin-bonded inlay-retained and wing-retained fixed partial dentures over 5 years

**DOI:** 10.1007/s00784-024-06103-5

**Published:** 2024-12-13

**Authors:** Wolfgang Bömicke, Julius Haas, Sinclair Awounvo, Peter Rammelsberg, Stefan Rues

**Affiliations:** 1 Department of Prosthetic Dentistry, Medical Faculty, Heidelberg University, Heidelberg, Germany; 2 Department of Orthodontics, Ludwig-Maximilians-University, Munich, Germany; 3 Institute for Medical Biometry, Medical Faculty, Heidelberg University, Heidelberg, Germany; 4 Poliklinik für Zahnärztliche Prothetik, Im Neuenheimer Feld 400, 69120 Heidelberg, Germany

**Keywords:** Tooth wear, Dental restoration wear, Zirconium oxide, Ceramics, Dental enamel

## Abstract

**Objectives:**

To prospectively evaluate the wear of posterior zirconia resin-bonded fixed partial dentures (RBFPDs) with polished occlusal surfaces and their natural enamel antagonists compared to contralateral controls in an enamel-enamel contact over 5 years.

**Materials and methods:**

In six patients with either an inlay- or wing-retained RBFPD made of monolithic 3 mol% yttria-stabilized tetragonal zirconia polycrystal (3Y-TZP), wear was evaluated indirectly using baseline and annual polyvinyl siloxane impressions. Resulting gypsum models were digitized and aligned by unchanged surface areas. Wear was analyzed by depth and area. For each parameter, descriptive statistics were used to express the degree of wear observed at yearly intervals for each group. A linear mixed regression analysis was performed to compare the enamel opposing 3Y-TZP group and the enamel-enamel controls at tooth level. All statistical tests were conducted at the 5% significance level.

**Results:**

After 5 years, the mean enamel wear depth of teeth opposing 3Y-TZP was 77 μm, compared to 54 μm for control teeth. No wear was observed on the 3Y-TZP RBFPDs. Maximum enamel wear depth and wear area were 229 μm and 9 mm², respectively, for teeth opposing 3Y-TZP, and 135 μm and 5 mm² for control teeth. Significant differences in mean enamel wear depth emerged after 2 years of observation.

**Conclusions:**

Polished 3Y-TZP caused more enamel wear than natural antagonists over 5 years, but the wear remained within the range reported for other commonly used indirect restorative materials.

**Clinical relevance:**

The clinical use of polished 3Y-TZP restorations appears to be justified in terms of natural antagonist wear behavior.

## Introduction

A ceramic material that can be used for almost all indications in the restorative prosthetic spectrum has been developed in the form of 3 mol% yttria-stabilized tetragonal zirconia polycrystal (3Y-TZP) [1, 2]. First generation 3Y-TZP is characterized by high strength but is also very opaque, which is why it is usually veneered. Comparatively high chipping rates of the ceramic veneer prompted a series of design, processing, and material improvements, which also included attempts to increase the translucency of the material. By reducing the alumina content and increasing the sintering temperature, a modest improvement in translucency of 3Y-TZP was achieved while retaining the exclusively tetragonal crystal structure of the material [3]. The material used in this study is part of this next generation of 3Y-TZP materials. Restorations made of this material have been used clinically successfully both monolithically in the posterior region and with vestibular veneers in the so called esthetic zone, achieving high patient satisfaction with the esthetics of the restorations [4]. Newer generations of zirconia (ZrO_2_) use an increase in yttria content (4 mol%/5 mol%) and the associated partial stabilisation of the cubic crystal phase to increase the translucency of the material and thus improve esthetics, albeit at the expense of reduced bending strength [5].

One contributing factor to the popularity of ZrO_2_ as a restorative material is that it can be processed on a modern computer-aided design (CAD)/computer-aided manufacturing (CAM) workflow. In combination with digital impression-taking and data transfer, further efficiencies can be achieved in the restorative process chain, including chairside fabrication [6].

Despite the mechanical and processing advantages of ZrO_2_, its abrasiveness compared to other dental restorative materials has been questioned since its introduction to the dental field. Initial misconceptions about the relationship between hardness and abrasiveness of ceramic restorative materials were later dispelled by research [7, 8]. Rather than absolute hardness, the final surface treatment appears to be the determining factor for wear on the antagonist tooth [9]. Malkondu et al. [7] reviewed 12 in vitro studies regarding intrinsic and antagonist wear and concluded that polished ZrO_2_ restorations were the most wear-favourable compared to glazed or veneered ZrO_2_ restorations. However, it is well known that in vitro wear tests vary widely in design and validity and generally mimic oral cavity properties only to a limited extent [10]. So-called two-body wear methods are usually carried out using chewing simulators with a sliding phase during contact of antagonist and test specimen. With the Minnesota method, it is assumed that 250,000 in vitro load cycles with 13.35 N force magnitude correspond to about 1 year in vivo, whereas the Zurich method with 1,200,000 in vitro load cycles at 49 N force magnitude is meant to be equivalent to 5 years of clinical wear [11]. In the three-body wear test (e.g. ACTA method), two contacting wheels with different speeds on their circumferential surfaces are placed in a slurry with abrasive potential. Different study groups use different media like millet, poppy seeds, or silica particles used in toothpastes making comparison of results from different studies difficult. In addition, the degree of wear also depends on the individual tooth and restoration morphology, which is not considered in tests with flat test specimens against natural teeth [10, 12]. Finally, in vitro tests execute in general uniaxial sliding movements and can only partially or inadequately simulate the complex kinematics during chewing in the oral cavity. This underlines the importance of clinical studies to generate meaningful wear data for restorative materials and the natural antagonist [13].

Clinical studies of ZrO_2_ restorations have produced conflicting results regarding antagonist wear due to methodological differences. While one study reported less wear compared to enamel [14], others found more wear [15–18] or no significant difference [19]. Variability results from different evaluation methods, such as focusing only on occlusal contact areas (OCAs) [16], overall surface wear [19], or neglecting surface differentiation [15, 18]. Sample sizes have mostly been small (10 to 20 participants) with short study durations (6 months to 2 years studies) [14–19]. Pathan et al. included 60 participants but lacked a control group, unlike other studies using split-mouth designs [14].

Given the lack of data, it seemed reasonable to conduct a longitudinal clinical study that specifically evaluated the antagonist regions of interest (ROIs) over a longer period than previous studies to investigate the extent to which ZrO_2_ restorations made of 3Y-TZP wear the natural antagonist over time and how these results compare to wear between two natural antagonists.

The aim of this study was, therefore, to prospectively evaluate wear of 3Y-TZP restorations and their natural enamel antagonists in comparison to the wear at enamel-enamel contacts measured on a contralateral natural antagonist pair over a period of 5 years. To assess the differences in wear performance, it was hypothesized that there would be no statistically significant differences in wear between enamel antagonists, antagonists of a monolithic 3Y-TZP restauration and the restauration itself.

## Materials and methods

The wear evaluation was planned as a secondary endpoint of a monocentric, prospective, randomized clinical trial designed to observe the clinical performance of ceramic inlay-retained and wing-retained resin-bonded fixed partial dentures (RBFPDs). The study protocol was approved by the local ethics committee (registration number S-083/2013) and pre-registered on clinicaltrials.gov (registration number NCT01997710).

More detailed information on the clinical and laboratory procedures used in the study and methodological aspects related to the primary objective (complication-free survival of RBFPDs) can be found elsewhere [20].

A total of 30 participants received either one inlay-retained or one wing-retained 3-unit RBFPD for replacement of a missing second premolar, first molar, or second molar, fabricated monolithically from 3Y-TZP (Cercon ht; DeguDent GmbH, Hanau, Germany). The material was individually stained (Colour Liquid Prettau; ZirkonZahn GmbH, Gais, Italy) in the pre-sintered state and stained and glazed (Cercon stain/glaze; Dentsply Sirona) after sintering at 1,500 °C (Cercon heat plus; DeguDent GmbH, total sintering time: 7:35 h, sintering curve: room temperature > 22 °C/min > 900 °C > 11 °C/min > 1,500 °C/2:25 h > allow to cool to 200 °C, firing chamber closed). The restorations were adhesively cemented (Panavia 21 TC; Kuraray Europe GmbH, Hattersheim am Main, Germany).

During clinical try-in, and in some cases also after adhesive cementation, all RBFPDs were adjusted in the area of the occlusal contacts using ceramic-specific diamond rotary instruments (ZR6881.314.016 and ZR8881.315.016, Gebr. Brasseler; Lemgo, Germany) and then polished to a high gloss (Zenostar polishing kit, Wieland Dental; Pforzheim, Germany), resulting in polished (glaze layer completely removed) restoration surfaces for wear evaluation.

The main inclusion criteria for wear evaluation were patients with both natural antagonists without crowns or large fillings opposing the RBFPD (enamel-3Y-TZP contact) and a pair of occluding contralateral natural teeth (enamel-enamel contact) serving as controls. A total of 18 participants did not meet these criteria and were excluded from wear measurement. An additional 3 participants were excluded because they were not available for the entire 5-year study period. In addition, 2 patients had to be excluded because their baseline impressions (taken 2 weeks after RBFPD placement) were considered erroneous. Finally, 1 participant had to be excluded because the teeth were iatrogenically altered during the study period. Thus, 6 participants were included in the wear evaluation (Table 1). The clinical characterization of the study participants included sociodemographic data, RBFPD type and location, and the results of a bruxism screening consisting of a self-report of bruxism activity using the bruxing scale [21] (the bruxing scale results from the answers to 4 questions regarding teeth clenching or grinding during the day and at night; each question has 5 ordinal answer options from 0 = never, 1 = sometimes, 2 = regularly, 3 = frequently to 4 = always, as well as the option of not answering a question; the highest individual value of the 4 questions results in the bruxing scale), tooth wear based on the attrition score [22] (0 = no grinding facets, 1 = minimal wear of cusps or incisal edges, 2 = grinding facets parallel to the normal contour, 3 = recognizable flattening of cusps or incisal edges, 4 = complete loss of contour and exposed dentin) assessed clinically for the prospective RBFPD abutments, the presence of cheek and/or tongue impressions, and the measurement of nocturnal bruxism activity using a single-use electromyography device [23] (BiteStrip, up2dent.com pixeltown oHG; Pulheim-Stommeln, Germany; L = no bruxism or minor bruxism corresponds to up to 30 episodes within 5 h in the sleep laboratory, 1 = mild bruxism: corresponds to 31 to 60 episodes within 5 h in the sleep laboratory, 2 = moderately severe bruxism: corresponds to 61 to 100 episodes within 5 h in the sleep laboratory, 3 = severe bruxism: corresponds to more than 100 episodes within 5 h in the sleep laboratory, E = error: incorrect measurement, - = no muscle activity measurable or no skin conductivity: in 2% of the population, the skin is not suitable for this test).

**Table 1:** Sociodemographic and clinical baseline data of the study participants

No.	Attrition score	Bite strip	Cheek impressions	Tongue impressions	Bruxing scale (sum score)	Age [years]	Sex	RBFPD type	Teeth* examined for wear depending on contact situation	
									enamel- 3Y-TZP	enamel-enamel
1	1	2	No	No	1	34.68	M	W	15	25, 35
2	2	L	No	No	0	59.17	M	W	15	25, 35
3	0	L	No	No	0	43.62	M	W	35	15, 45
4	0	L	No	No	2	39.63	M	I	35	15, 45
5	1	1	Yes	No	0	63.71	W	I	15, 16	25, 35, 26, 36
6	0	L	Yes	Yes	2	57.59	M	W	35, 37	15, 45, 17, 47

Attrition score (0–4) [22]: 0 = no grinding facets, 1 = minimal wear of cusps or incisal edges, 2 = grinding facets parallel to the normal contour, 3 = recognizable flattening of cusps or incisal edges, 4 = complete loss of contour and exposed dentin; Bite strip: Bruxism screening via single-use electromyography device (BiteStrip, up2dent.com pixel-town oHG; Pulheim-Stommeln, Germany) [23]: L = no bruxism or minor bruxism corresponds to up to 30 episodes within 5 h in the sleep laboratory, 1 = mild bruxism: corresponds to 31 to 60 episodes within 5 h in the sleep laboratory, 2 = moderately severe bruxism: corresponds to 61 to 100 episodes within 5 h in the sleep laboratory, 3 = severe bruxism: corresponds to more than 100 episodes within 5 h in the sleep laboratory, E = error: incorrect measurement, - = No muscle activity measurable or no skin conductivity: in 2% of the population, the skin is not suitable for this test. Bruxing scale (0–4) [21]: The bruxing scale results from the answers to 4 questions regarding teeth clenching or grinding during the day and at night. Each question has 5 ordinal answer options from 0 = never, 1 = sometimes, 2 = regularly, 3 = frequently to 4 = always, as well as the option of not answering a question. The highest individual value of the 4 questions results in the bruxing scale. Deviating from this definition, a sum score was calculated, as no bruxing scale score > 1 was expected due to the exclusion criteria of the underlying clinical trial. W = wing-retained RBFPD, I = inlay-retained RBFPD, *FDI notation

A positive self-report of grinding or clenching on the bruxing scale (score > 1 in response to one of the 4 questions included), an attrition score > grade 2, and severe bruxism diagnosed by the electromyography device led to exclusion from the underlying clinical prognostic study of RBFPDs. The use of an occlusal splint was documented.

**Fig. 1:**
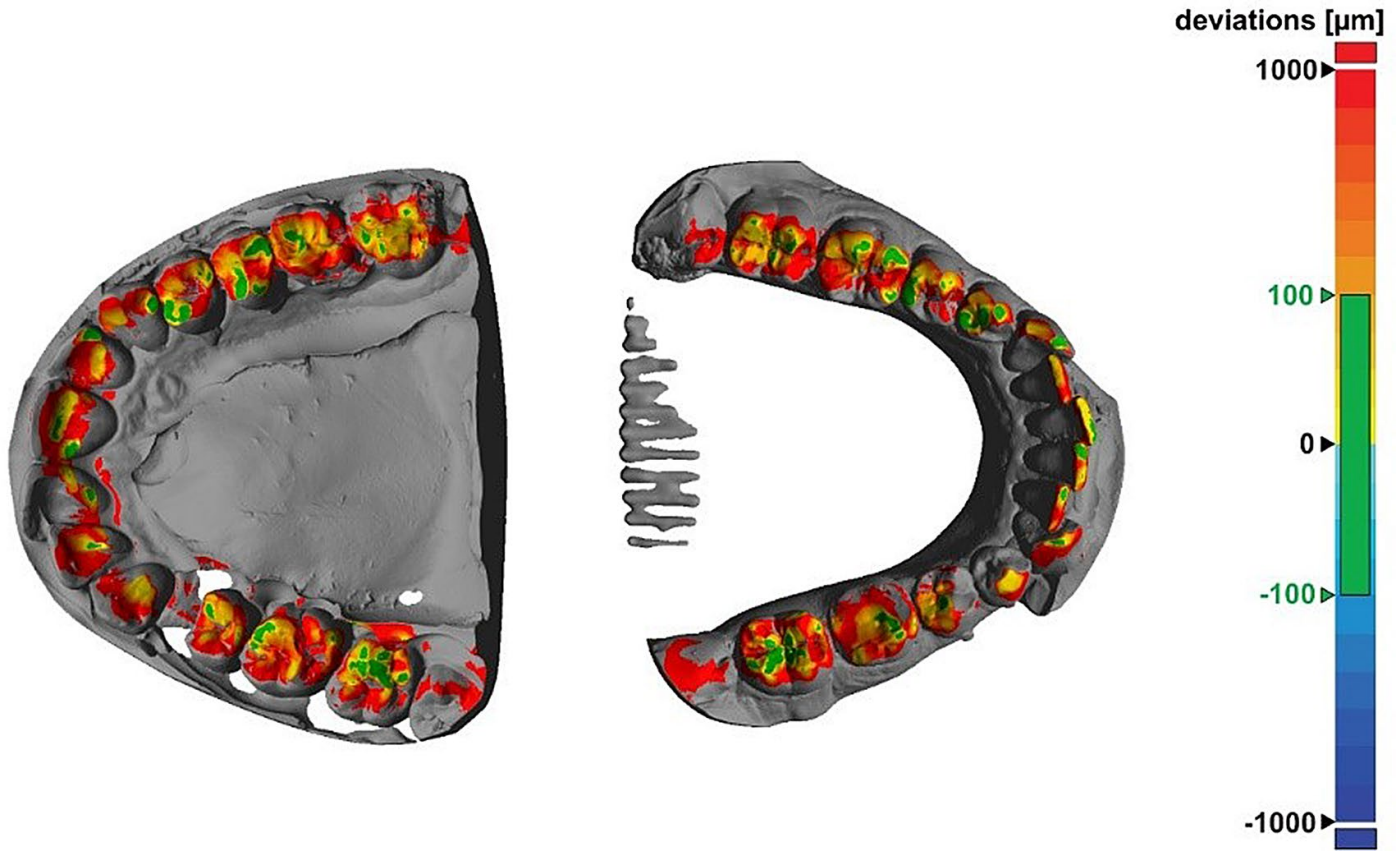
Graphical representation of the distances between the upper (left) and lower (right) jaw. Contact points appear green in the 100 μm tolerance range

For wear evaluation, dual-phase single step polyvinyl siloxane impressions (Flexitime heavy tray in combination with Flexitime Correct Flow; Kulzer GmbH, Hanau, Germany) were made at baseline and annual follow-up visits. Intraoral photographs with occlusal contacts marked with 8 μm colored occlusal foil (Bausch, Cologne, Germany) were used as a control for the contact areas identified from the digitized models as well as the regions of interest (ROIs) identified in the subsequent wear evaluation. Models resulting from pouring the impressions with Type IV dental gypsum (GC Fujirock EP; GC Europe NV, Leuven, Belgium) were digitized with a laboratory scanner (D2000; 3Shape A/S, Copenhagen, Denmark) using quality control software (Convince 2015; 3Shape). Surfaces were triangulated homogeneously with a triangle edge length of 60 μm and exported as stl-files. Additionally, the models were scanned in maximum intercuspation. After best-fit alignment of each individual jaw scan with the intercuspation scan (Geomagic Design X; 3D Systems, Rock Hill, USA), the distances between the maxilla and mandible were graphically displayed as false-colour plots and the occlusal contact areas were associated with inter-jaw distances of less than 100 μm. Figure 1 shows an example of a false-color plot with a 100 μm threshold in green, highlighting the identified contact areas.

Three different wear groups were defined: (1) wear of polished 3Y-TZP in contact with natural enamel (3Y-TZP RBFPD), (2) wear of natural enamel in contact with polished 3Y-TZP (natural tooth opposing 3Y-TZP RBFPD), and (3) natural enamel in contact with natural enamel (occluding contralateral natural teeth, controls).

**Fig. 2:**
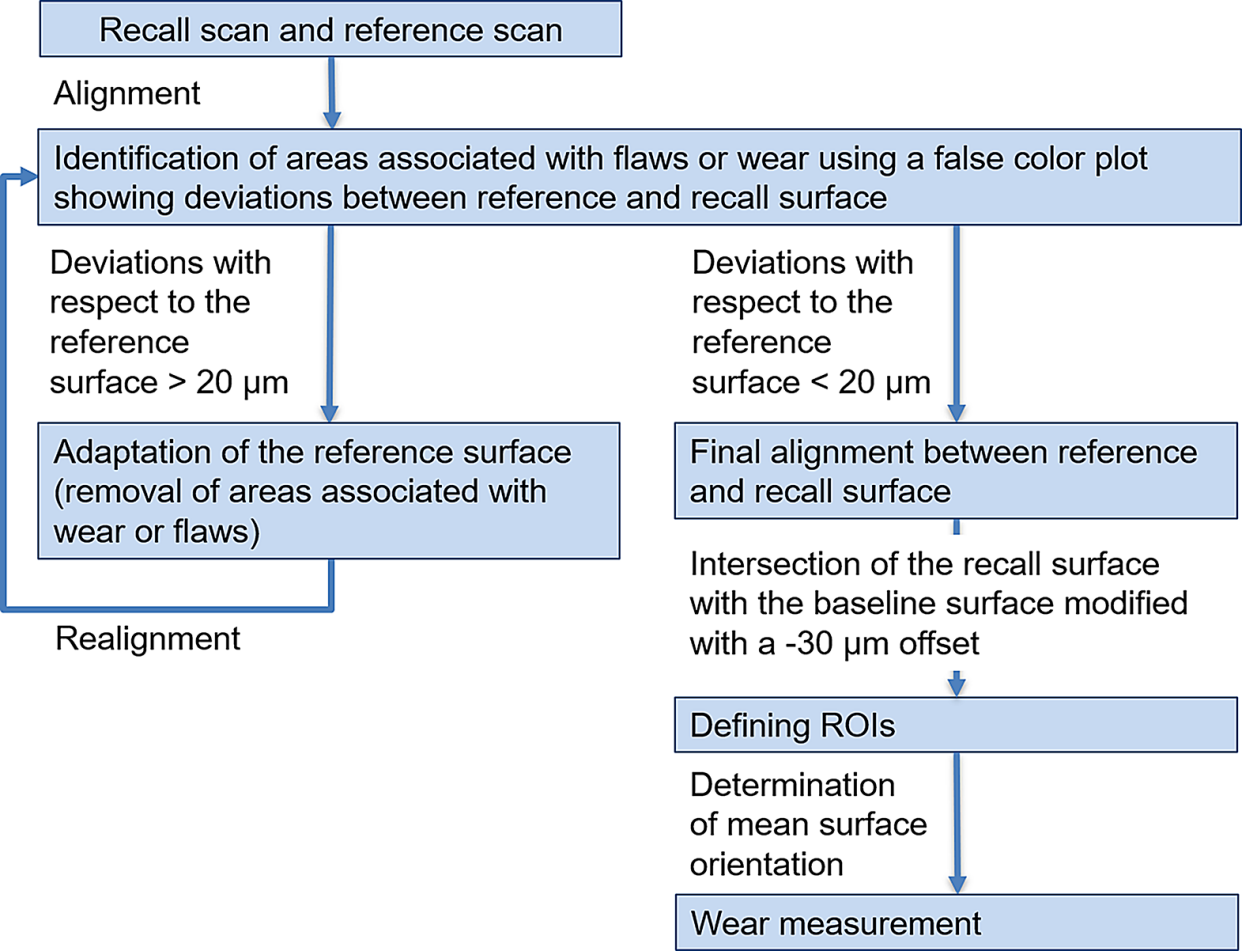
Methodological flowchart describing working sequences from model scan to final wear measurement

**Fig. 3:**
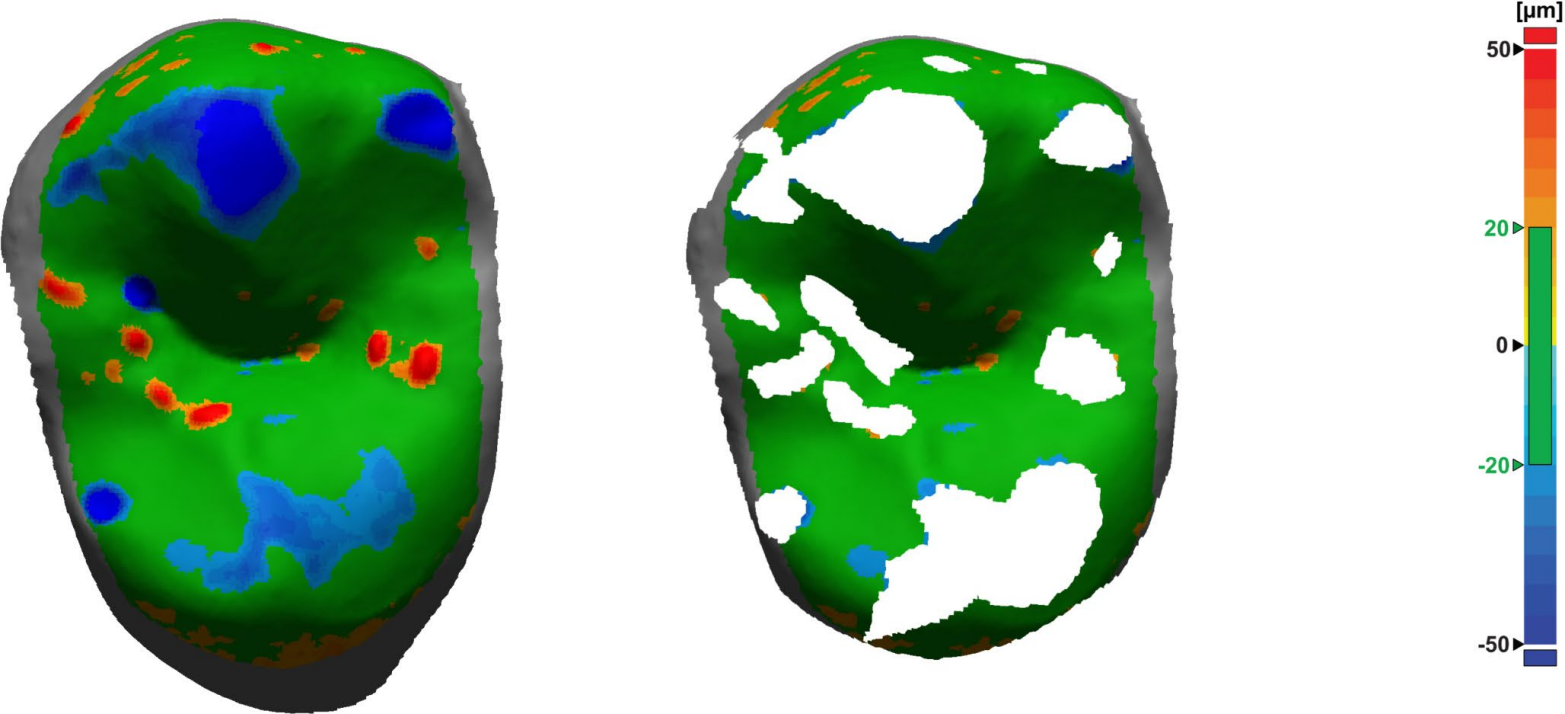
Surface deviations after initial alignment of baseline and recall situation (left). Reference surface used for final alignment after iterative removal of inaccuracies and areas of wear (right). Green areas lie within tolerance range of 20 μm; red: positive deviations correlating with impression errors, blue: negative deviations correlating with abrasion or model errors (e.g. fissures that flowed out differently or bubbles in gypsum model)

In a first step, teeth included in the wear evaluation were individually separated from each model scan. To enable wear evaluation, each follow-up scan was aligned with the baseline situation (Fig. 2). This critical process had to be performed manually to get the best possible result and was performed by a single operator (JH) after prior training. For the alignment of the baseline and follow-up scans (Geomagic Design X), only those surface areas that did not change over time were used. In consequence, these reference areas had to be iteratively identified by using a copy of the occlusal surface of the baseline scan (reference surface) and stepwise (re-)alignment of the recall surface and removal of regions correlating with wear or flaws from the reference surface. Since during the first alignment flawed or worn surface regions are present and the alignment is not ideal, only areas showing large deviations or obvious flaws were removed and the alignment process repeated (Fig. 3). This iterative sequence of reference surface adaptation and realignment was repeated until all distance deviations between reference surface and follow-up scan were beneath a threshold of 20 μm. Based on a pilot study, this 20-µm threshold for clinical studies was the optimal value being small enough that meaningful wear evaluation could be carried out and large enough that omnipresent inaccuracies (impression taking, gypsum cast, scanning inaccuracies) did not lead to excessive reference surface adaptation as well as inconsistent or incorrect ROI identification.

**Fig. 4:**
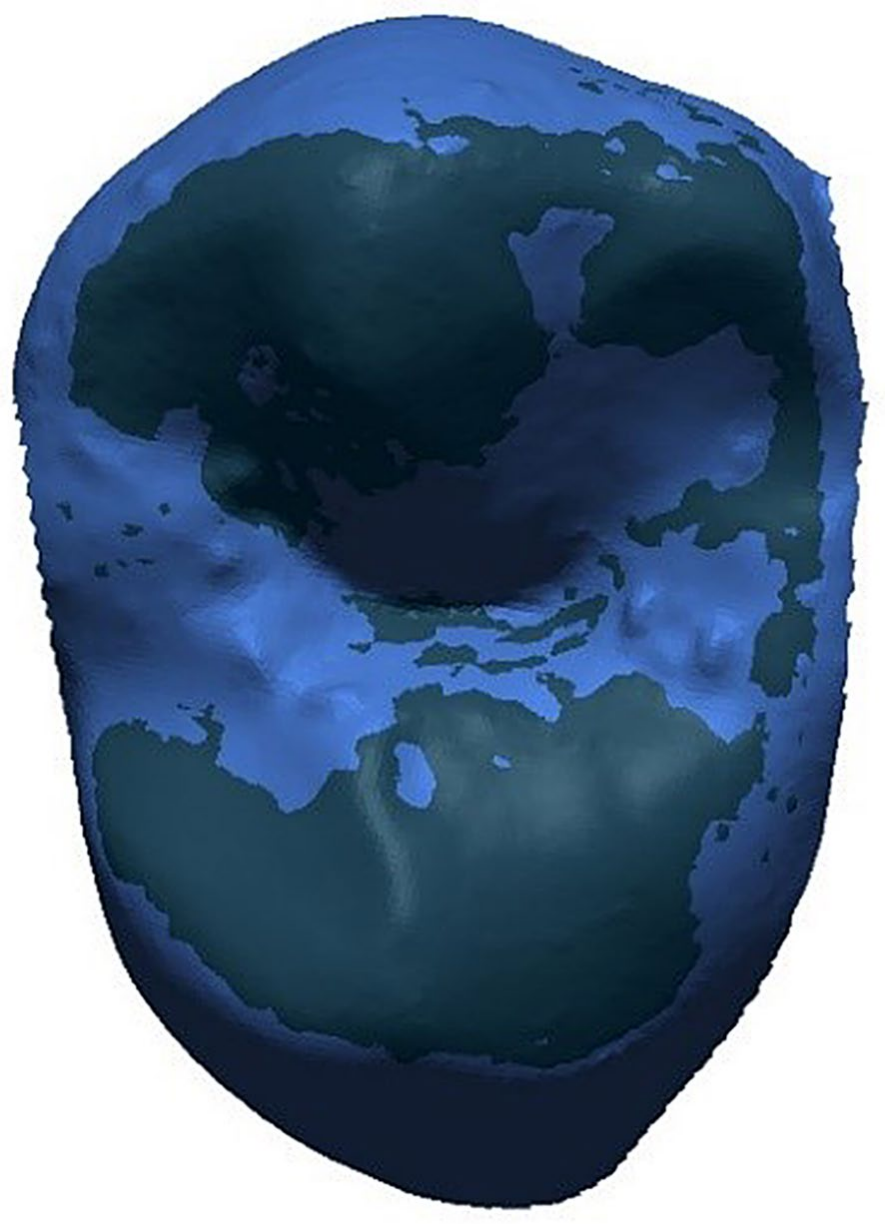
Baseline surface (dark blue) and recall surface (light blue) in final alignment position. No clear regions of interest for wear measurements detectable

**Fig. 5:**
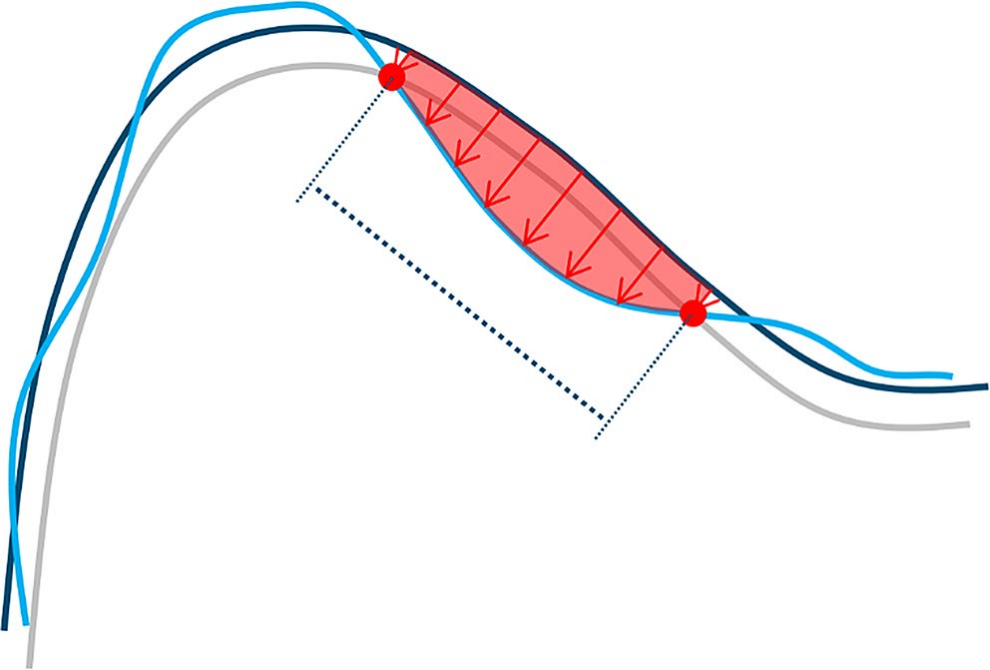
Two-dimensional scheme illustrating the wear measurement methodology: -30 μm offset (gray) of baseline surface (dark blue) shows clear intersection (red dots) with recall surface (light blue). Measurement direction for wear depth (red arrows) was defined by normal vector of best fitting plane to offset surface within respective ROI. Wear area projected into best-fitting plane of ROI (indicated by dotted line) was assessed. Wear volume (space between baseline and recall surface below ROI, indicated in red) can be calculated as product of mean vertical depth and wear area

**Fig. 6:**
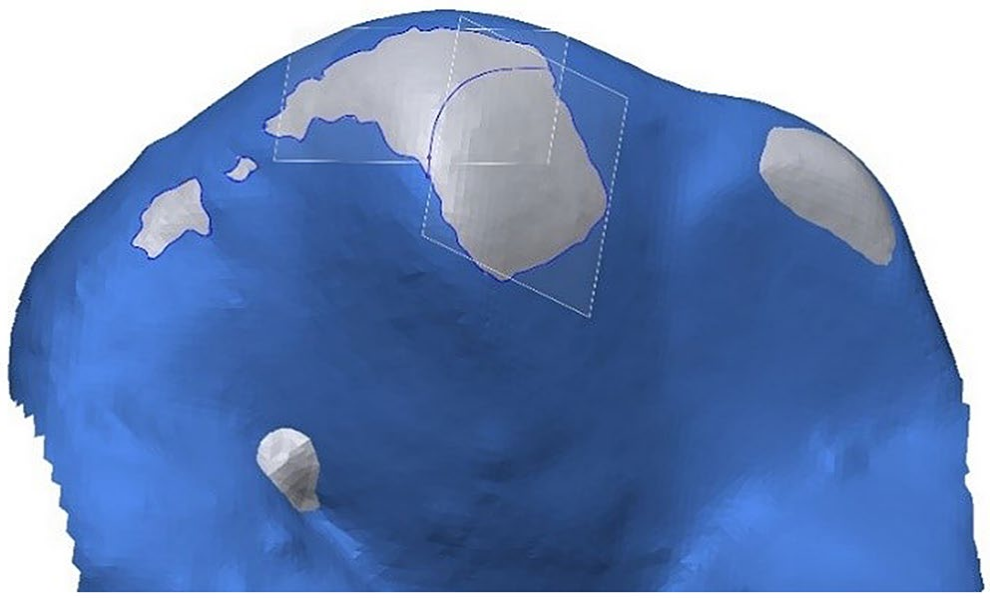
Clear identification of regions of interest (ROIs) associated with wear, delineated by the offset surface (gray) exposed above the recall surface (light blue). ROIs were split along cusp ridges (blue line) to create separate, more planar ROIs and correspondingly separate best fitting planes for wear measurement

After final superimposition (Fig. 4), a clean demarcation of wear areas (regions of interest, ROIs) was achieved by intersecting a slightly modified baseline tooth geometry (-30 μm offset oriented into the tooth volume) with the follow-up scan (Figs. 5 and 6). Again, the -30 μm offset parameter (along with the 20 μm alignment threshold) was the smallest value at which meaningful results could be generated based on clinically obtained dental impressions.

For each ROI, a constant projection direction was defined by the normal vector of a plane best fitted to the offset surface (representing the baseline surface orientation) within the respective ROI (Fig. 5). Thus, wear was always measured approximately perpendicular to the orientation of the baseline surface. For large ROIs with deviations in local surface orientation of more than approximately 30°, the ROIs were subdivided into smaller, more planar ROIs (Fig. 6). In addition, ROIs in the marginal ridge area had to be delineated because they extended into the proximal space. For this purpose, a boundary line was defined that was the same for all recalls.

Deviations between the baseline and the recall surface were measured orthogonally to the mean surface orientation (best fitted plane) at each point of the previously defined 30 μm measurement grid. The wear measurement itself was performed analogously to the previously used wear analysis system Scan3D/Match3D (Straumann CAD/CAM, Gräfeling, Germany) [24] using Matlab (Matlab, Mathworks Inc, Natick, USA). Wear is associated with loss of material and is therefore measured by negative values. The largest deviation represented the maximum wear depth and the mean value of all measurements (weighted with the size of the associated projected ROI area) represented the mean wear depth. For wear area, the size of the projected ROI area was used. In this methodology, the product of (projected) wear area and mean wear depth equals wear volume (not evaluated in this report).

For statistical analysis, tooth-based values were generated. Consequently, wear values for each tooth were derived from the wear values of the ROIs determined for the respective tooth. The wear area of all involved ROIs could be simply summed up. The maximum wear depth was the maximum wear depth found for all ROIs. The mean wear depth was calculated as sum of all mean values of the involved ROIs weighted with their respective wear area proportion.

The magnitude of the various wear parameters in each group for each recall year were described with the number of missing values, mean value, standard deviation, median, minimum, and maximum. Furthermore, box and whisker plots were used to graphically represent the maximum and mean wear depths. For each recall year, the wear parameters were evaluated individually using a linear mixed regression analysis. All models included the group (enamel-enamel vs. enamel-3Y-TZP) as fixed factor and the patient as random factor. Effect size and associated 95% confidence intervals and p-values were calculated. In addition, linear regression graphs were calculated for maximum and mean wear depths, showing the evolution of wear in a chronological context. Due to the exploratory nature of the study, all p-values were interpreted strictly descriptively. All statistical tests were conducted at the 5% significance level. All analyses were conducted using the statistical software R v4.3.3 (The R Project for Statistical Computing, www.r-project.org) and IBM SPSS Statistics v28 (IBM Corp, Armonk, New York, United States).

## Results

The sociodemographic data of the study participants, the results of the bruxism screening, and the evaluated teeth are shown in Table 1. Only one participant was categorized with moderately severe bruxism using the portable electromyography device, while the other patients had lower scores. None of the participants wore an occlusal splint.

Identified ROIs were consistent without exception up to the 4-year follow-up, i.e., a ROI at any time contained the ROI of the previous recall. At the 5-year follow-up, however, some wear areas were inconsistent, as (almost) unchanged surface areas for scan alignment became increasingly rare.

There was no measurable wear of the 3Y-TZP RBFPDs in contact with enamel even after 5 years, so this contact group was not included in the statistics.

**Fig. 7:**
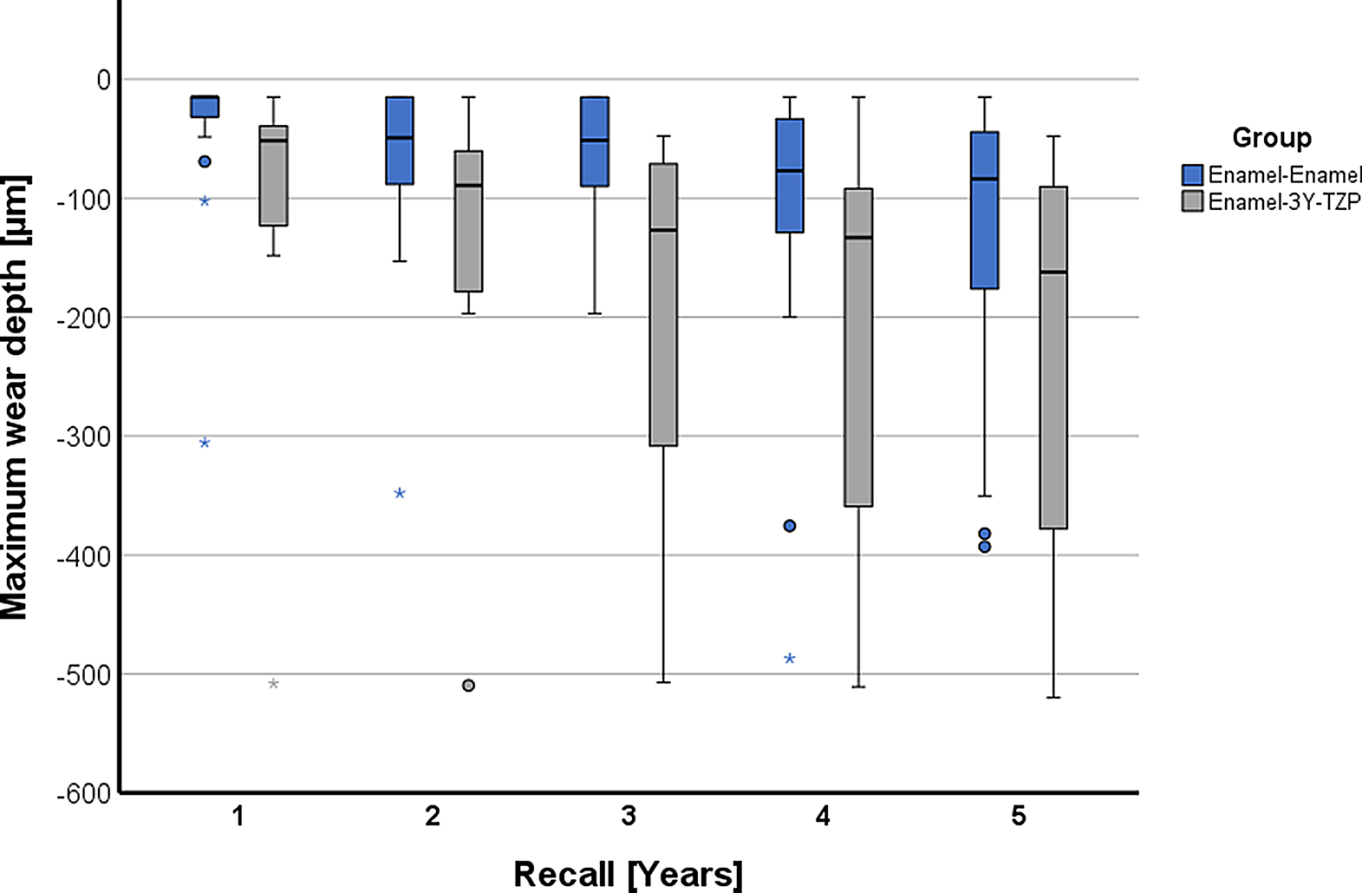
Grouped box and whisker plot for maximum wear depth over time

**Fig. 8:**
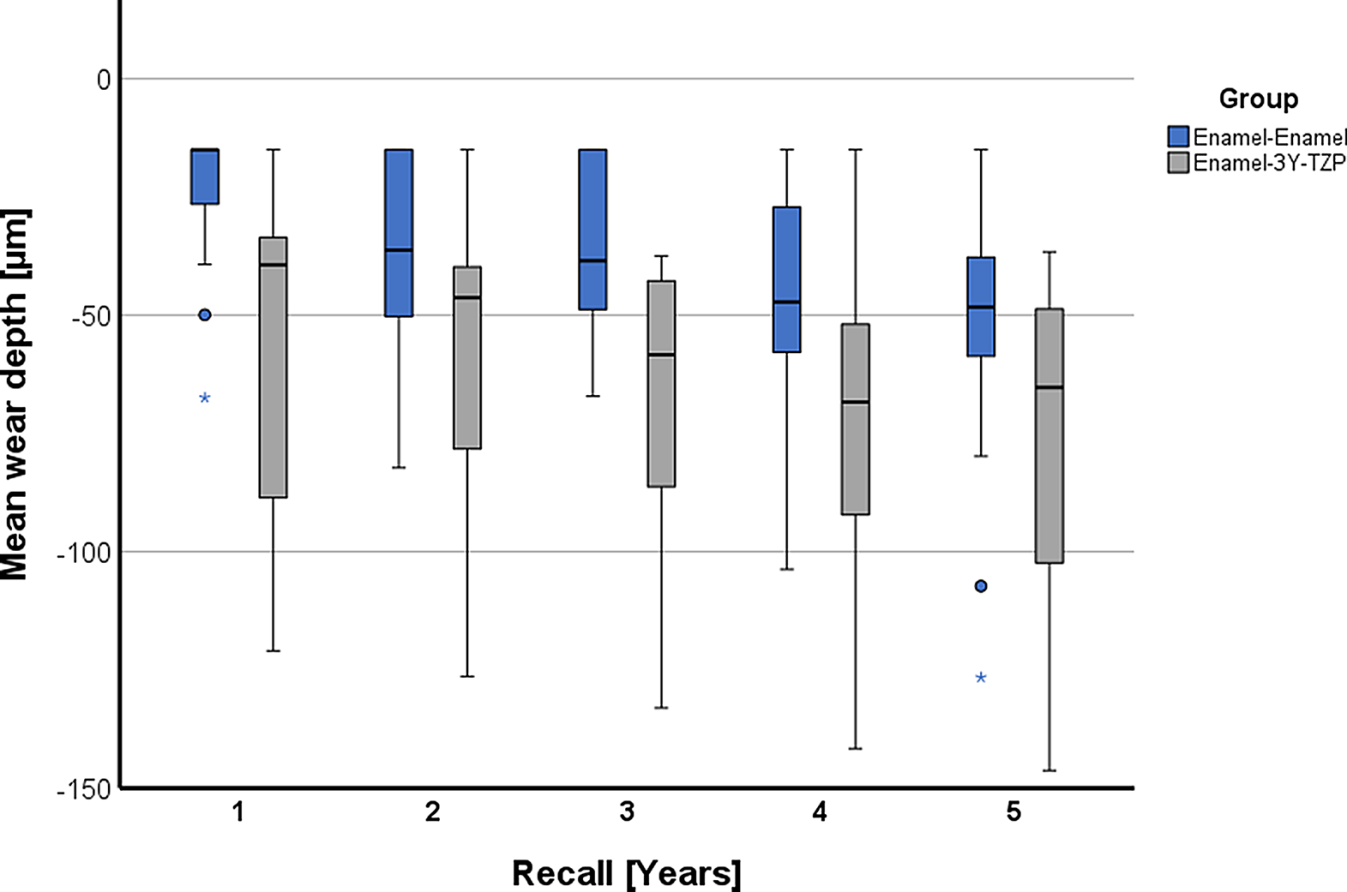
Grouped box and whisker plot for mean wear depth over time

The results for the variables maximum wear depth, mean wear depth, and wear area for years 1 to 5 are listed in Table 2 and graphically presented in Fig. 7 for maximum wear depth and in Fig. 8 for mean wear depth.

**Table 2:** Summary of maximum wear depth, mean wear depth, and wear area at 1 to 5 years for the enamel-enamel controls and the enamel opposing 3Y-TZP. In contrast to the number of teeth evaluated for each group (n), N gives the total number of teeth for which the respective wear data was available

Recall	Teeth	Variables	Enamel-Enamel	Enamel-3Y-TZP
[years]	n		(*n* = 16)	(*n* = 8)
1	24	Maximum wear depth [µm]		
		N	16	8
		Mean (SD)	-44 (74)	-119 (163)
		Median (IQR)	-15 (-23, -15)	-52 (-111, -39)
		Range	-305, -15	-508, -15
		Mean wear depth [µm]		
		N	16	6
		Mean (SD)	-23 (16)	-57 (41)
		Median (IQR)	-15 (-21, -15)	-39 (-72, -34)
		Range	-67, -15	-121, -15
		Wear area [mm²]		
		N	4	7
		Mean (SD)	4.3 (6.3)	2.0 (3.6)
		Median (IQR)	1.5 (0.8, 5.0)	0.2 (0.1, 1.8)
		Range	0.4, 13.7	0.1, 10.0
2	24	Maximum wear depth [µm]		
		N	16	8
		Mean (SD)	-74 (83)	-148 (157)
		Median (IQR)	-49 (-85, -15)	-89 (-169, -64)
		Range	-348, -15	-510, -15
		Mean wear depth [µm]		
		N	16	8
		Mean (SD)	-39 (22)	-59 (34)
		Median (IQR)	-36 (-49, -15)	-46 (-76, -40)
		Range	-82, -15	-126, -15
		Wear area [mm²]		
		N	11	7
		Mean (SD)	3.6 (5.9)	3.5 (4.9)
		Median (IQR)	0.9 (0.3, 3.0)	1.9 (0.7, 3.2)
		Range	0.1, 18.3	0.2, 14.1
3	24	Maximum wear depth [µm]		
		N	16	8
		Mean (SD)	-64 (51)	-196 (164)
		Median (IQR)	-51 (-85, -15)	-127 (-291, -77)
		Range	-197, -15	-507, -48
		Mean wear depth [µm]		
		N	16	8
		Mean (SD)	-36 (17)	-68 (35)
		Median (IQR)	-38 (-48, -15)	-58 (-74, -43)
		Range	-67, -15	-133, -38
		Wear area [mm²]		
		N	11	8
		Mean (SD)	2.3 (2.4)	6.0 (6.8)
		Median (IQR)	1.1 (0.6, 3.1)	2.5 (1.2, 9.9)
		Range	0.1, 6.7	0.3, 18.5
4	24	Maximum wear depth [µm]		
		N	16	8
		Mean (SD)	-120 (133)	-212 (175)
		Median (IQR)	-77 (-120, -42)	-133 (-353, -103)
		Range	-487, -15	-511, -15
		Mean wear depth [µm]		
		N	16	8
		Mean (SD)	-48 (26)	-73 (39)
		Median (IQR)	-47 (-58, -33)	-68 (-82, -55)
		Range	-104, -15	-142, -15
		Wear area [mm²]		
		N	12	7
		Mean (SD)	4.2 (4.6)	8.8 (9.4)
		Median (IQR)	2.6 (1.5, 4.3)	4.1 (1.5, 15.4)
		Range	0.3, 15.6	0.6, 22.9
5	24	Maximum wear depth [µm]		
		N	16	8
		Mean (SD)	-135 (129)	-229 (174)
		Median (IQR)	-84 (-161, -47)	-162 (-378, -102)
		Range	-393, -15	-520, -48
		Mean wear depth [µm]		
		N	16	8
		Mean (SD)	-54 (30)	-77 (38)
		Median (IQR)	48 (-56, -40)	-65 (-94, -51)
		Range	-127, -15	-146, -37
		Wear area [mm²]		
		N	14	8
		Mean (SD)	5 (6)	9 (10)
		Median (IQR)	4 (1, 6)	4 (2, 17)
		Range	0, 21	0, 24

SD = Standard deviation, IQR = Interquartile range

The maximum wear depth after 5 years was -229 ± 174 μm for enamel opposing 3Y-TZP and -135 ± 129 μm for enamel-enamel controls.

The mean wear depth after 5 years for enamel opposing 3Y-TZP was -77 ± 38 μm, whereas it was -54 ± 30 μm for enamel-enamel controls.

The mean wear area after 5 years was 9 ± 10 mm^2^ for enamel opposing 3Y-TZP and 5 ± 6 mm^2^ for enamel-enamel controls.

Statistical analysis of the data did not show a consistently significant difference between the enamel-3Y-TZP group and the enamel-enamel controls but did indicate greater wear in the 3Y-TZP antagonists for the parameters maximum wear depth and mean wear depth (Table 3). This was reflected in statistically significantly higher values for the 3Y-TZP antagonists for the parameter maximum wear depth in years 3 and 4 (*p* < 0.043) and for the mean wear depth almost over the entire study period (*p* < 0.045), with the latter parameter failing to reach the significance threshold at the 2-year recall (*p* = 0.051). For the parameter wear area the groups only significantly differed at the 3-year time point (*p* = 0.031).

**Table 3:** Annual comparison between enamel-enamel controls and enamel opposing 3Y-TZP using linear-mixed regression analysis

	Maximum wear depth [µm]			Mean wear depth [µm]			Wear area [mm²]		
Recall	Estimate	95% CI	p-Value	Estimate	95% CI	p-Value	Estimate	95% CI	p-Value
1	-75	-174, 24	0.130	-34	-58, -10	0.008	-2.3	-8.9, 4.4	0.500
2	-73	-174, 28	0.150	-19	-39, 0.13	0.051	-0.14	-5.8, 5.5	> 0,900
3	-132	-205, -59	0.001	-32	-48, -16	< 0,001	4.5	0.49, 8.5	0.031
4	-92	-181, -3.3	0.043	-25	-48, -2.6	0.031	5.0	-1.5, 11	0.120
5	-94	-189, 2.1	0.055	-23	-46, -0.61	0.045	4.0	-2.5, 10	0.200

**Fig. 9:**
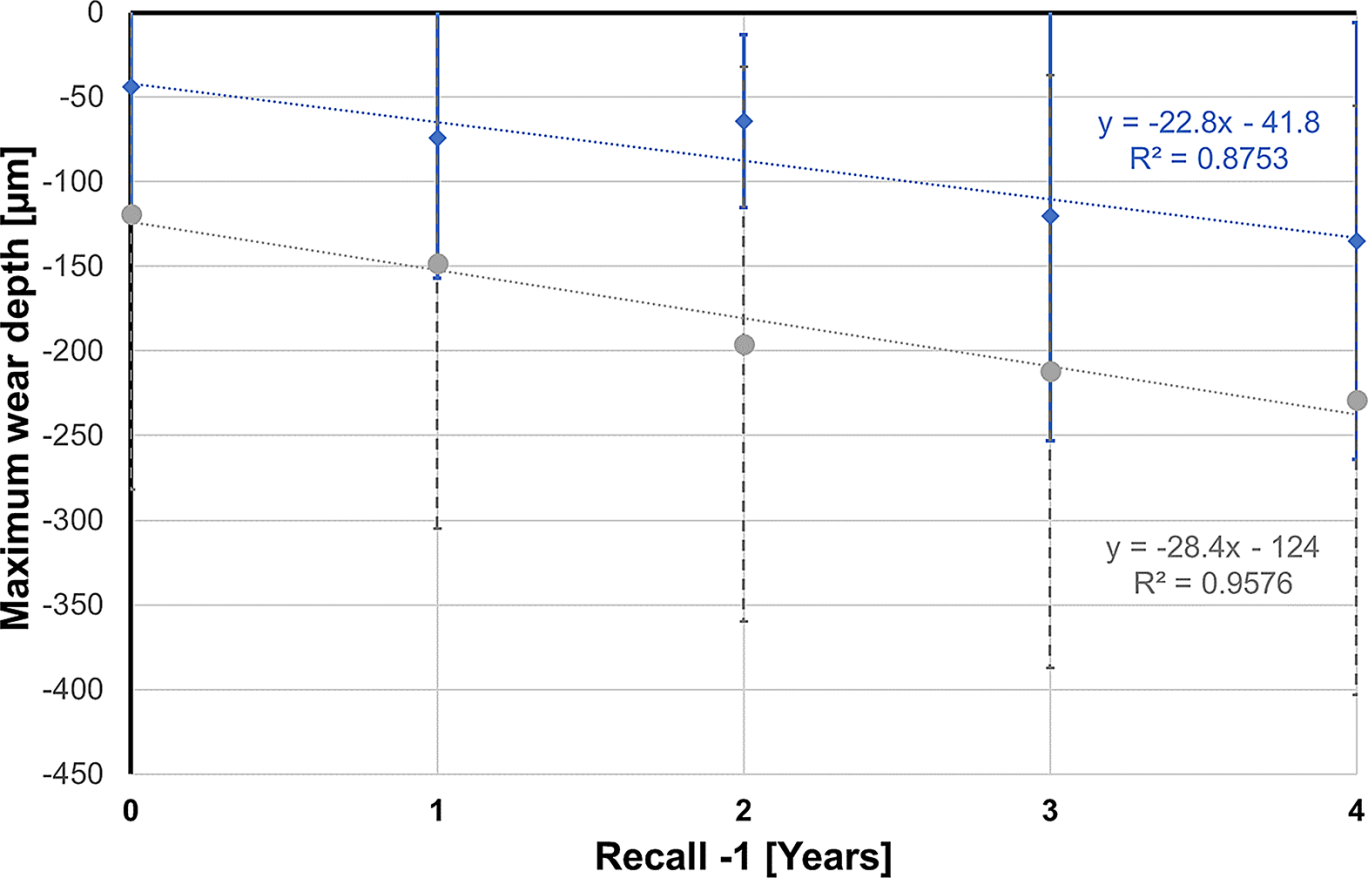
Regression lines for maximum wear depth over time (blue: enamel-enamel group; gray: enamel-3Y-TZP group). With 1-year recall as starting point (time measured as Recall -1), constant gives maximum wear depth at 1-year recall and regression coefficient represents annual increase

**Fig. 10:**
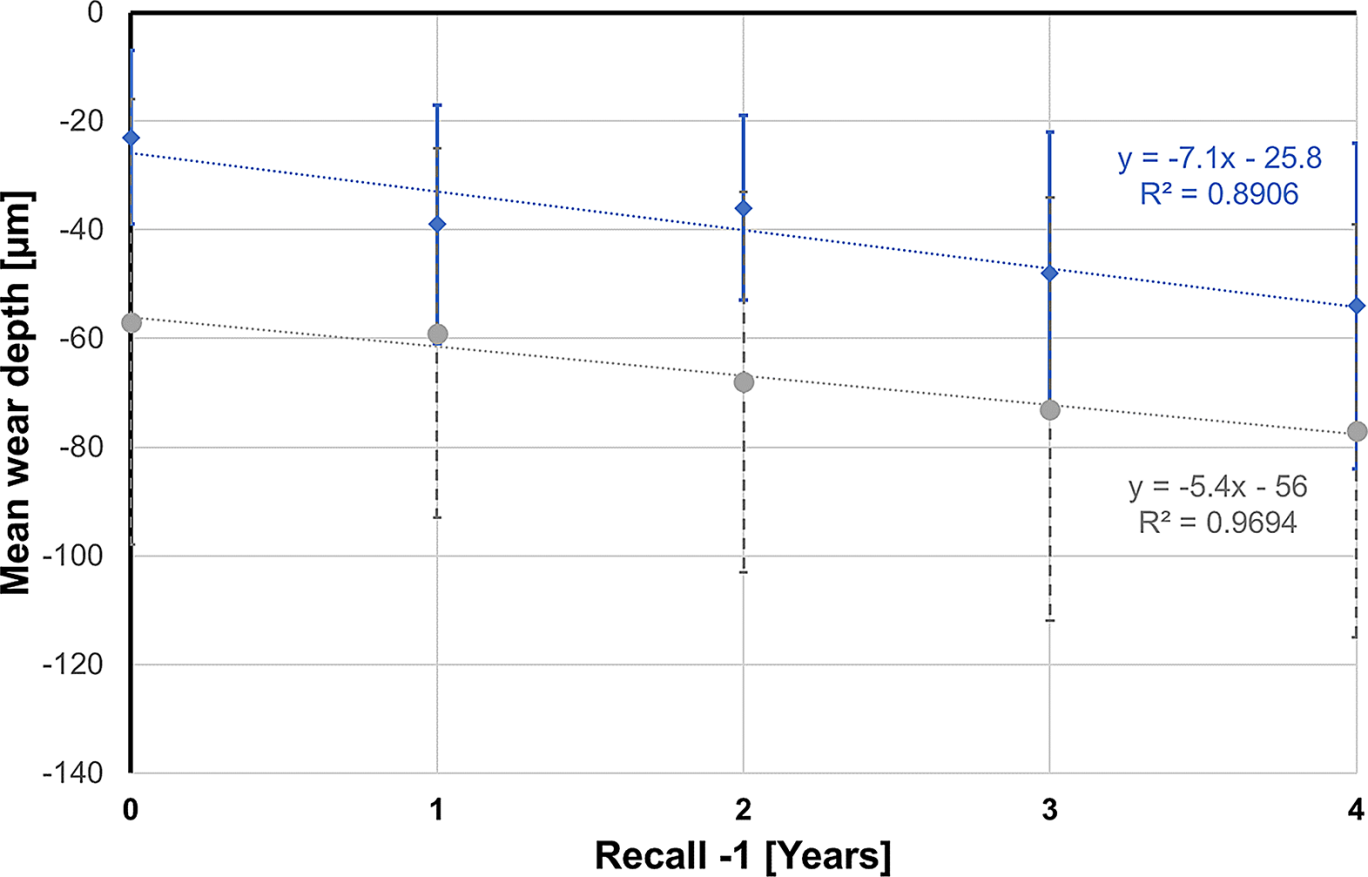
Regression lines for mean wear depth over time (blue: enamel-enamel group; gray: enamel-3Y-TZP group). With 1-year recall as starting point (time measured as Recall -1), constant gives mean wear depth at 1-year recall and regression coefficient represents annual increase

In purely descriptive terms, the wear behavior between the groups appears to be different, especially in the first year, as can be seen from the almost parallel regression lines for the maximum wear depth (Fig. 9) and the mean wear depth (Fig. 10), which take the wear of the first year as the starting point. Their slope indicates an annual increase in the maximum wear depth of 28.4 μm in the enamel-3Y-TZP group and 22.8 μm in the enamel-enamel group. The corresponding values for the mean wear depth are 5.4 μm (enamel-3Y-TZP) and 7.1 μm (enamel-enamel).

## Discussion

Based on the results, the initial hypothesis was partially rejected, and a statistical test between wear of the 3Y-TZP RBFPDs and the other two study groups could not be performed due to the lack of detectable wear on the ceramic material. When comparing the enamel opposing 3Y-TZP group with the enamel-enamel contact group, no consistent statistically significant difference could be found for the various wear criteria, but it became apparent that the 3Y-TZP opposing enamel group showed higher amounts of wear than the enamel-enamel controls. Observing the wear of the restoration and the wear of the antagonist plays a central role in the use of modern restorative materials in minimally invasive patient care. Accordingly, following the widespread introduction of monolithic 3Y-TZP into dental practice, a series of in vitro studies were conducted on this topic [7, 10, 11]. Apart from the result that the polished 3Y-TZP surface behaves most favourably towards the antagonist, these studies were unable to provide information about the actual clinical wear of 3Y-TZP and its antagonists.

For this reason, in vivo studies have been performed in the past to evaluate clinical wear over periods of 12 to 24 months [14, 15, 17, 19, 25, 26]. Considering that the survival probabilities of monolithic 3Y-TZP restorations are very promising [2, 20], the aim of this study was to investigate the wear behavior of these restorations over a longer period of time, exceeding 2 years.

For the above-mentioned shorter periods of time, the mean wear depth for enamel opposing 3Y-TZP was found in a range of 16 μm to 127 μm after 1 year [14, 15, 18, 25–29], which was comparable to 57 μm in this study. For the same observation period 23 μm were found for the enamel-enamel controls in this study, which also concurs with findings from the literature of 13 μm to 61 μm [15, 18, 25–28]. Also for a longer observation period of 2 years, the amount of wear observed here (59 μm for enamel opposing 3Y-TZP and 39 μm for enamel-enamel controls) is within the range found in other studies, as shown by comparison with Stober et al. [30] who reported 46 μm for enamel opposing 3Y-TZP and 19 μm to 26 μm for the enamel-enamel controls.

The 5-year results presented in this study showed no consistent statistically significant difference between the enamel opposing 3Y-TZP group and the enamel-enamel control group. However, due to the small number of study participants and the high variance of individual values in some of them, it can be expected that this difference will become more significant as the number of cases increases. Therefore, it is important to note that the enamel opposing 3Y-TZP group had higher wear values than the enamel-enamel control group at each observation time. This difference in wear was particularly large in the first year, which supports the theory of a so-called running-in wear period and is in agreement with the findings of Lambrechts et al. [31], Mehl et al. [32], and Esquivel-Upshaw et al. [33], who also described increased early antagonist wear in their studies. Over the remaining study period (years 2 to 5), a linear increase in mean wear depth was observed in both groups.

In a recent systematic review by Mao et al. [34], the enamel wear of various dental ceramic systems was investigated with follow-up periods of up to 24 months. Based on the included studies, lithium disilicate ceramic (mean wear depth: 5 μm) was considered superior to 3Y-TZP (mean wear depth: 40 μm) in terms of antagonist wear behavior, and metal-ceramic systems (mean wear depth: 83 μm) were considered inferior to 3Y-TZP, regardless of ZrO_2_ surface treatment. It was also concluded that a polished 3Y-TZP surface (mean wear depth: 39 μm) caused less antagonistic enamel wear than a glazed surface (mean wear depth: 63 μm). In their discussion, the authors also addressed the common finding of other studies [35–38] that antagonistic 3Y-TZP has been reported to cause less enamel wear than feldspathic porcelain. Considering that the results of Mao et al. were limited to periods of up to 24 months, the results of the present study, with a mean wear depth of the natural antagonist of 77 μm after 5 years, indicate that polished 3Y-TZP appears to perform very well clinically in a longer-term view. This is an important finding. Since initial studies on the prognosis of monolithic restorations made of 3Y-TZP have shown that the use of the material is associated with comparatively low technical complication rates and that thin (minimally invasive) restorations with acceptable esthetics can be fabricated from the material [4], it can be assumed that monolithic 3Y-TZP will be increasingly used in the future, at least in the posterior region. The results of the present study therefore appear to support the clinical applicability of the restorative material, also regarding its antagonistic wear behavior over a longer period of time.

Methodologically, this study used the indirect method of wear measurement by means of a precision impression and a gypsum model, which was subsequently digitized. This is a well-established method used in all the in vivo studies mentioned before [14–19, 25, 26, 28, 33, 39]. When taking conventional impressions, the impression material inevitably tears in the area of FPD pontics and proximal spaces (if not blocked out) during removal. This was also the case in the present study, which is why the ROIs in marginal ridge areas had to be demarcated manually.

However, the accuracy of conventional impressions is not dependent on the surface being modelled. Clinical experience has shown that scanning high gloss polished zirconia is difficult and sometimes scanning powder is required. With a powder thickness of 20 μm to 40 μm [25] it is not practical to examine wear after scanning with powder. Nevertheless, it is done [40–42]. However, scanning technology is constantly evolving. Meanwhile, Schlenz et al. [43, 44] have shown in both an in vitro [43] and an in vivo [44] study that intraoral scanners can be superior to conventional impressions, particularly in imaging proximal spaces. The ability to accurately record such areas appears to be advantageous, as they are then available as unaltered areas for a matching process. In this context, the study of Schlenz et al. [45] did not use scan powder but just dried the teeth.

Schlenz et al. [45], Marro et al. [46] and Bronkhorst et al. [42] investigated the wear of enamel-enamel contacts over periods of 2 to 5 years using the superimposition of intraoral scans. While Schlenz et al. [45] found lower maximum wear depth after 2 years, Marro et al. [46] and Bronkhorst et al. [42] found higher mean wear depth after 2 and 5 years compared to the findings in this study. Esquivel-Upshaw et al. [47], on the other hand, examined 3Y-TZP restorations and their antagonists for wear using intraoral scans over a 12-month period and came up with a mean 3Y-TZP antagonist wear value of 55 μm. This result is within the range of the previously highlighted other in vivo studies and this study [14, 15, 18, 25, 26, 28, 33, 39].

Since inaccuracies in digitized dental arches tend to increase with longer spans for both techniques, intraoral scanning and classic impression taking, wear is typically evaluated on a single tooth basis [32, 41] as done in this investigation to achieve best possible alignment. In addition, meaningful alignment has to be based on unchanged surface areas. This is a critical aspect for wear evaluation because suboptimal alignment can change the calculated wear values significantly. In this study, the finding of the reference surface areas was done manually in an iterative process. In future, AI tools may help to further automize wear evaluation. A new aspect of the current approach is the automatic ROI detection which is in contrast to the previously used method [32] using manual ROI selection. If no ROI definition takes place, only the maximum wear depth can be defined unambiguously, while mean wear or wear area cannot be given in a meaningful way [41, 42].

In the previous wear evaluations used by the authors [32, 48], wear was measured in vertical direction. As long as the worn surfaces were placed rather horizontally in the 3D scanner, this method makes sense. Problems arise, however, for inclined or curved surfaces: For geometric reasons, the measurement error then exceeds 15% from an angle of 30° from the normal vector. Therefore, one ROI-specific measurement direction was used, and ROIs were subdivided if surface orientation of the baseline scan varied by more than 30°. To omit a change in measurement direction for any ROI over time, the measurement direction was only determined based on the last recall and used for all preceding recalls. Other wear evaluations just using surface to surface distance measurement in CAD or quality control software [41, 42] face the problem that distances are measured perpendicular to the target surface (recall surface!). In consequence, measurement directions at any given position along the worn tooth surface can change significantly from one recall to the next, especially if the worn surface shows regions with high curvature.

When comparing deviations of digitized gypsum casts based on polyvinyl siloxane impressions with a reference model in vitro [49], both trueness and precision were < 6 to 11 μm on a single tooth level. This already includes the manufacturer’s specified accuracy of < 8 μm of the laboratory scanner (D2000, 3Shape A/S). During wear evaluation, two such flawed cast scans must be aligned on a single tooth basis and deviation of < 10 to 20 μm will occur even for regions showing no changes. With in vitro wear measurements, reliable ROI detection could be performed with an offset surface at 20 μm distance from the baseline surface. Lohbauer and Reich [19] for example described in their in vivo study all measurements lower than 25 μm as artifacts. Especially when working with digitized data on a single tooth level, intraoral scans may be an alternative to classic impression taking in future studies. Witecy et al. [50] describe the error for in vitro intraoral scanning as 10 μm to 15 μm per scan. If scan powder must be used, however, this easily multiplies. When working with clinical data, impression accuracy is a little bit worse than that found in vitro and a 30 μm offset value had to be chosen for reliable ROI detection. Surface deviations outside the ROIs were interpreted as random deviations or artifacts and therefore excluded from evaluation. This blind spot for wear smaller than 30 μm seems acceptable [19] and the alignment accuracy of this study was at least as accurate as reported in previous studies. Regarding the inaccuracies of classic precision impressions as well as intraoral scanning, it is at least questionable how accurate numbers below 30 μm in other studies are. On this behalf it also is not surprising, that this study was not able to measure any wear of the 3Y-TZP RBFPDs. Nevertheless, after 1 year Selvaraj et al. [18] and Tang et al. [25] managed to determine mean wear depth of 3Y-TZP of 13 μm to 27 μm. Stober et al. [17] found 14 μm of mean wear depth of 3Y-TZP after two years. Those numbers are all slightly under the detection threshold of the present study. The observed problem of decreasing alignment accuracy over time leading to sporadic inconsistencies in detected wear areas after 5 years, can be attributed to (small) changes of the entire tooth surface (e.g., erosion, tooth brushing). Thus, one is faced with the problem that reliable reference surfaces are associated with increasing inaccuracies or are no longer available after a very long observation time. For further investigation, regarding the decreasing reference surface areas over time, the question can certainly be raised as to what extent wear measurements can be relied on for longer observation periods.

The study has several other limitations. No ex vivo roughness measurements were performed to provide a quantifiable result of the roughness achieved on the surfaces under evaluation. The study protocol, however, required a smooth, high-gloss surface comparable to enamel after occlusal adjustment. This was confirmed by two independent clinical evaluators who assigned an FDI score of 1 (clinically excellent/very good) to the restorations in the study for the criterion of surface lustre and texture after placement [51].

Despite the long observation period, this study was certainly limited by the small number of patients. This is a general problem of in vivo wear examination. All studies using a split-mouth design need natural enamel controls in one half of the mouth and an enamel surfaced tooth opposing the investigated material in the other half of the mouth. Consequently, a large number of patients has to be excluded, because they cannot match this criterion. However, this is the only study design suitable for in vivo wear measurement. A central role in the genesis of individual wear are habit, diet, and musculature activity [12]. This differs from patient to patient. By being limited to smaller numbers of study participants in a clinical trial versus in vitro studies this method can compensate those individual effects in comparison to a literature control group.

However, it is also known, that in addition to individual differences in the development of wear, inter-individual differences also play a major role in the development of wear and tear. Schierz et al. [52] showed for a German study population of 836 people that the factors position of the teeth in the dental arch, age, jaw, and sex influenced the level of tooth wear in a descending order. Of particular importance was that a more anterior position of the tooth is associated with more wear. Due to our small patient population, such inter-individual differences could not be considered, which is reflected in the large variances of the individual measured values at the different measurement times. Viewed longitudinally, however, these inter-individual differences are the same for each measurement time point and are therefore of minor relevance for the longitudinal assessment in terms of wear development. However, it should be noted that this study was purely exploratory and was conducted as part of a pilot study. It therefore provides an indication of the range of possible values for the wear parameters of interest. The information obtained may be useful in a future confirmatory analysis.

Finally, the number of patients excluded show how frequently a wide variety of restorative materials act as antagonists in the patient’s mouth. Wear behaviour of different restauration materials as antagonists to each other has hardly been investigated, at least in vivo. This leads to another limitation of this study and simultaneously highlights the need for future research. The results are for 3Y-TZP, which means that they cannot be automatically extrapolated to the new (partially cubic) zirconia materials.

## Conclusion

Antagonistic wear of polished 3Y-TZP restorations was higher than that of control teeth over a 5-year observation period, but within the range reported for other commonly used indirect restorative materials.

## Data Availability

The data on which the results of this study are based can be made available upon reasoned request to the corresponding author.
